# The effects of sex and age on movie-watching functional connectivity and movie clip classification

**DOI:** 10.1007/s00429-025-02962-0

**Published:** 2025-07-09

**Authors:** Chengxiao Yang, Bharat B. Biswal, Pan Wang

**Affiliations:** 1 MOE Key Laboratory for Neuroinformatics, Center for Information in Medicine, School of Life Science and Technology, The Clinical Hospital of Chengdu Brain Science Institute, University of Electronic Science and Technology of China, Chengdu, 611731 China; 2 Department of Biomedical Engineering, New Jersey Institute of Technology, Newark, NJ 07102 USA

**Keywords:** Average cross-session correlation, Demographic effect, Functional connectivity, Movie watching, Support vector machine

## Abstract

Functional connectivity (FC) is a key tool for understanding the complex interactions within the human brain, highlighting connections between various regions. This study delves into the multifaceted influences shaping functional magnetic resonance imaging FC patterns during movie watching, focusing on the effects of sex, age, and movie clip. Leveraging the Human Connectome Project dataset, we systematically examine FC patterns elicited during movie watching. Notably, sex-specific variations in FC are observed, with females exhibiting heightened FC within visual, limbic, and default mode networks, while males display predominant intra-network connectivity within somatomotor and attention networks. Age-related variations further manifest, revealing FC increases with age in early adulthood (21–35 years old) within some specific networks. Moreover, our investigation unveils the profound influence of movie clips on FC patterns, with significant interactions observed between clips, sex, and age. Feature selection using the Average Cross-Session Correlation method highlights FC as distinct fingerprints of clips, and the Support Vector Machine classifier shows high accuracy (Accuracy > 0.9) when using these features. Our findings underscore the importance of considering individual demographic factors and external stimuli in understanding neural connectivity dynamics during movie-watching, with implications for both basic neuroscience research and clinical neuroimaging applications.

**Supplementary Information:**

The online version contains supplementary material available at 10.1007/s00429-025-02962-0.

## Introduction

Regions within the brain interconnect and influence each other rather than operating as isolated islands (Stern 2022). Functional connectivity (FC), reflecting the intricate statistical dependencies between brain regions, provides the possibility to investigate the coordinated activity among different brain regions and deeply understand the functional organization of the human brain (Friston 1994). Although several investigations have illuminated the richness of physiological and psychological insights embedded within FC, establishing its relevance to development (Betzel et al. 2014; Zuo et al. 2017), cognitive functions (e.g., attention, executive control, memory, etc.) (Dong et al. 2015; Jeong et al. 2015; Rohr et al. 2017), and psychiatric disorders (e.g., major depressive disorder, bipolar disorder, attention deficit hyperactivity disorder, etc.) (Gong et al. 2019; Shen et al. 2015; Tang et al. 2018), the confounding influences of demographic factors on it are often overlooked.

Previous studies have demonstrated that FC patterns are influenced by multiple factors, notably sex and age, which reflect the nuanced individual differences in neural connectivity architecture (Biswal et al. 2010). Resting-state functional magnetic resonance imaging (rsfMRI) studies reported that sex-specific variations in FC have been noted (Frigerio et al. 2021). Specifically, females exhibited heightened FC within the Default mode network (DMN), spanning the posterior precuneus/cingulate, bilateral inferior parietal cortex, and medial prefrontal cortex (Bluhm et al. 2008). In contrast, males demonstrated predominant short-range FC in the right hemisphere (Tomasi and Volkow 2012), along with notable intra-network connectivity in females compared to males’ pronounced inter-network connectivity (Zhang et al. 2018). Similarly, the effect of age on FC has been widely reported, and DMN exhibited the most sensitivity to age-related changes (Bluhm et al. 2008; Campbell et al. 2013). Specifically, FC was observed to decrease in elders across not only the DMN but also within the dorsal attention network (DAN), sensorimotor network (SMN), visual network (VN), and fronto-parietal network (FPN) (Betzel et al. 2014; Cassady et al. 2019). Conversely, researchers observed elevated FC within the basal ganglia network in older individuals (Allen et al. 2011). Moreover, Tian and colleagues demonstrated a decline in intra-network connectivity and an increase in inter-network connectivity with advancing age (Tian et al. 2016). FC patterns are not solely determined by intrinsic factors but are also modulated by external stimuli. Stimulus-driven changes in FC have been observed across diverse experimental paradigms, highlighting the brain’s adaptive response to environmental inputs (Cisler et al. 2014). However, the nuanced effects of these factors, particularly during movie-watching experiences, remain poorly understood.

Naturalistic fMRI has a distinct advantage compared to resting and conventional task-based states (Di et al. 2022; Di and Biswal 2020; Ao et al. 2024). For instance, the naturalistic paradigm elicits intricate cognitive activity owing to the abundance of audio-visual natural stimuli, positioning itself in closer alignment with the complexity of real-life experiences compared to either of the states above (Vanderwal et al. 2019; Eickhoff et al. 2020). Movie watching, a prevalent conduit for naturalistic stimuli, has been widely utilized in cognitive neuroscience research. Studies have shown that the consistency of FC during movie-watching is comparable to or even greater than that observed during the resting state (Tian et al. 2021; Hu et al. 2023). This consistency may be affected by novelty effects during repeated movie viewings (O’Connor et al. 2017) or variations in movie selection across scans (Shearer et al. 2025). Moreover, leveraging these movie-evoked FC features has shown promise in enhancing the prediction of various individual characteristics, including age (Bi et al. 2024), behavior (Finn and Bandettini 2021), cognitive functions, and brain activity (Gal et al. 2022; Guan et al. 2023). Despite the increasing adoption of movies as stimuli in neuroscience investigations (Fenerci et al. 2024; Mittal et al. 2024; Watson and Andrews 2024; Xiao et al. 2024), the nuanced effects of sex and age on movie-evoked FC have remained inadequately understood. Furthermore, the influence of stimulus content on shaping FC patterns during movie-watching and whether these patterns can serve as predictive features for distinguishing between movie clips remains largely unexplored. Bridging these gaps in knowledge is imperative for fostering a comprehensive understanding of the neural substrates underlying movie-watching and their modulation by individual demographic variables.

Motivated by these considerations, this study investigated the effects of sex, age, and movie clip on FC patterns elicited during movie watching. Leveraging the Human Connectome Project (HCP) dataset, we systematically examined the respective influence of these factors on FC. Additionally, we utilized Support Vector Machine (SVM) models to capture the complex, multifaceted relationships within FC. This approach aimed to extend the utility of FC beyond traditional demographic and behavioral markers, positioning it as a fingerprint for movie clips and thereby deepening our understanding of the neural mechanisms involved in movie-watching experiences.

## Materials and methods

### Dataset

**Fig. 1:**
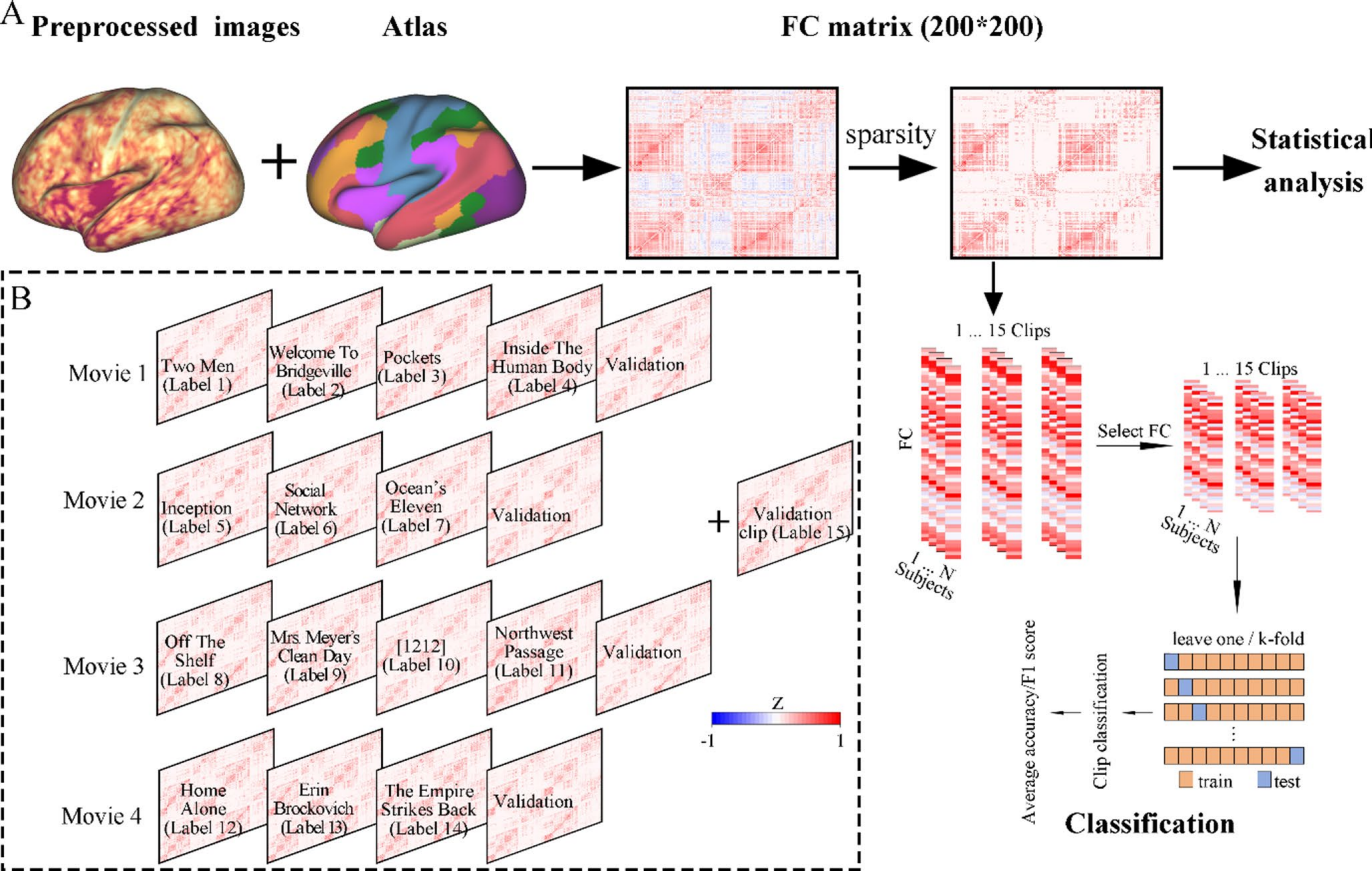
Procedure of analyzing. (**A**) Initially, regions of interest (ROIs) are delineated within the preprocessed image using a standardized template. Subsequently, pairs of ROI signals are systematically combined to compute Pearson correlation coefficients, yielding a FC matrix and an adjacency matrix is generated by selecting sparsity thresholds of each subject. The resulting adjacency matrix is then utilized for subsequent statistical analyses and machine learning classifications. (**B**) Average FC of the 15 clips with their coded labels in the 4 movie-watching runs; in particular, the validation clip was taken from the average of the four runs

The dataset utilized in this study was obtained from an unrestricted subset of the HCP release, which encompassed a total of 184 subjects (Barch et al. 2013). Each subject underwent scanning sessions over two days, acquiring rest state and movie-watching state fMRI data. During REST runs, subjects were instructed to maintain wakefulness and fixate on a crosshair. During Movie runs, subjects were required to engage in an audiovisual experience, viewing four or five movie clips with a 20-second rest interval between each clip. 171 out of 184 participants completed all required scans successfully in this study. The subjects were then categorized into three age groups based on the unrestricted release; however, two participants did not fit into these categories and were excluded from further analysis, resulting in a final cohort of 169 individuals eligible for subsequent evaluation (21–35 years old, 68 females). For the four movie-watching runs, we extracted the entire duration of each clip and its subsequent 10 TRs to account for hemodynamic peak delays (Siegel et al. 2016). Eventually, we got 15 clips of data for each subject (Fig. 1B). Additional details about the movie clips are provided in Table 1.

**Table 1:** Presentation order for human connectome project movie-watching data. TR: repetition time = 1000 Ms

Day 1				Day 2			
Movie 1		Movie 2		Movie 3		Movie 4	
Clip name	Duration	Clip name	Duration	Clip name	Duration	Clip name	Duration
Two Men	244 TR	Inception	227 TR	Off The Shelf	181 TR	Home Alone	233 TR
Welcome To Bridgeville	222 TR	Social Network	259 TR	Mrs. Meyer’s Clean Day	185 TR	Erin Brockovich	230 TR
Pockets	188 TR	Ocean’s Eleven	249 TR	[1212]	204 TR	The Empire Strikes Back	255 TR
Inside The Human Body	64 TR	Validation clip	83 TR	Northwest Passage	142 TR	Validation clip	83 TR
Validation clip	83 TR			Validation clip	83 TR		

### Imaging acquisition and preprocessing

fMRI data were collected using a 7T Siemens Magnetom scanner with a 32-channel head coil. All 7T fMRI data was acquired with a gradient-echo planar imaging sequence (repetition time = 1000 ms, echo time = 22.2 ms, flip angle = 45°, field of view = 208 × 208 mm, matrix = 130 × 130, number of slices = 85, 1.6 mm isotropic voxel resolution, multi-band factor = 5, image acceleration factor = 2, partial Fourier sample = 7/8, echo spacing = 0.64 ms, bandwidth = 1924 Hz/Px).

The movie fix-denoised preprocessed datasets were sourced from the HCP website (https://db.humanconnectome.org/). The data was preprocessed with the HCP pipeline. This standard processing includes motion correction, distortion correction, high-pass filtering (cutoff = 2000 s), nonlinear alignment to 2 mm MNI template space, and artifact removal procedure using FSL’s FIX. The head motion (along with 12 head motion confound parameters), respiration, and structured artifacts were regressed from the filtered data by the ICA + FIX processing (Glasser et al. 2013; Vu et al. 2017). Lastly, the preprocessed fMRI data for each participant were resampled onto a standardized cortical surface mesh representation (fs_LR 32k mesh).

### Functional connectivity analysis in naturalistic condition

Since the FC matrix in the study consists of nodes (brain regions) and edges (correlations between nodes), we first segmented the brain into 200 regions of interest (ROIs) under 7 networks using the Craddock parcellation (Craddock et al. 2012). Subsequently, ROI signals were extracted by averaging voxel signals within each parcel, and statistical dependencies were established through pairwise Pearson correlations among the 200 ROIs. To improve normality, the Fisher z-transformed values were applied to follow-up analysis (Fisher 1915). Specifically, the connectivity matrix of each subject was thresholded by a sparsity at 0.25 to reduce noisy connections and bias from threshold selection (Di et al. 2013; Ning et al. 2022; Pang et al. 2020). Further network analyses were based on each subject’s 200*200 thresholded FC matrix. Given the potential influence of sparsity selection on the current study, we extended our analysis to include additional sparsity values of 0.2 and 0.3.

### Statistical analysis

The computational analysis was conducted using custom MATLAB scripts. To assess the effects of sex, age, movie clip, and their interactions on FC, a linear mixed-effects model was employed. In this model, the movie clip was treated as a within-subjects factor, while sex and age were treated as between-subjects factors. Mean head motion was included as a covariate to control for potential confounding effects (Power et al. 2019). After identifying FCs with significant effects, we assigned them to the seven networks and calculated the number of FCs that were significantly stronger in either sex/age group. This allowed us to determine the directionality of the sex/age effect at the network level. We calculated partial eta squared (η_p_^2^) as a measure of effect size. False Discovery Rate (FDR) correction (*p* < 0.05) was applied to all statistical analyses with multiple comparisons. Specifically, we used the Benjamini-Hochberg procedure, which ranks the individual p-values in ascending order and calculates an adaptive threshold to determine statistical significance while controlling the proportion of false positives (Wilks 2006).

### Movie clip identification

Studies have demonstrated that FC contains valuable physiopsychological information, enabling the prediction of individual characteristics such as age, sex, and cognitive performance (Bi et al. 2024; Nakano et al. 2021; Zhang et al. 2018). In this study, we leveraged the robust capabilities of SVM algorithms to formulate a classifier model tailored for categorizing movie clips. We considered each of the 15 movie clips as independent categories for SVM classification, assigning them virtual labels (1, 2, 3,…, 15). The classifier employed the default parameters of MATLAB embedded functions *fitcecoc.* The leave-one-out cross-validation (LOOCV) approach was conducted. Specifically, the model was trained on N-1 subject data while tested on the remaining subjects.

Feature selection plays a crucial role in machine learning, offering a pathway to significantly enhance classification accuracy by discerningly choosing FC containing subject-specific information. In each LOOCV, we employed the Average Cross-Session Correlation (ACSC) score to select optimal FC features based on the train set to reduce redundancy and prevent overfitting. The ACSC was derived from a cost function determined by a cross-session similarity matrix (Peña-Gómez et al. 2018). Specifically, the upper triangular matrix was reshaped into vectors of length n*(n-1)/2 for each subject’s symmetric FC matrix (*n* = 200). For each subject, this process was independently performed in each of the 15 clips, resulting in a final set of 15 *FC{si}* vectors, where *si* represents the subjects. Subsequently, the Euclidean distance across all 168subjects (N-1, *N* = 169) and across 15 clips was computed to generate the similarity matrix *S* of size 168*168, where *S*_*i, j*_ denotes the cross-session distance between FC of the *i*^*th*^ subject and the *j*^*th*^ subject (i, j = 1, 2,… 168). The diagonal elements of S are expected to show the minimum distance, reflecting the similarity of each subject across sessions. The ACSC score was calculated by subtracting the average of off-diagonal elements from the average of diagonal elements in similarity matrix *S*. Large ACSC scores indicate low different-subject cross-session similarity but high same-subject cross-session similarity, thereby indicating the validity of features as FC fingerprints (Li et al. 2021).

We conducted feature selection by choosing the top k edges (of the FC) with the highest ACSC scores, where k varied from 1,000 to 19,000 with an interval of 3,000. The selected FC edges were used as discriminative features for classifying 15 distinct movie clips on the test set, each meticulously assigned virtual category labels ranging from 1 to 15. The accuracy and F1 score of the SVM classifier were measured based on predicted and truth labels. Additionally, we included full FC edges for comparison purposes, thus evaluating the performance of ACSC-based feature selection against utilizing all available edges. Given the potential for leave-one-out cross-validation to produce unstable and biased estimates, we further validated our results using 10-fold cross-validation to enhance the robustness of our findings (Varoquaux et al. 2017).

## Results

### Sex and age effects of functional connectivity

**Fig. 2:**
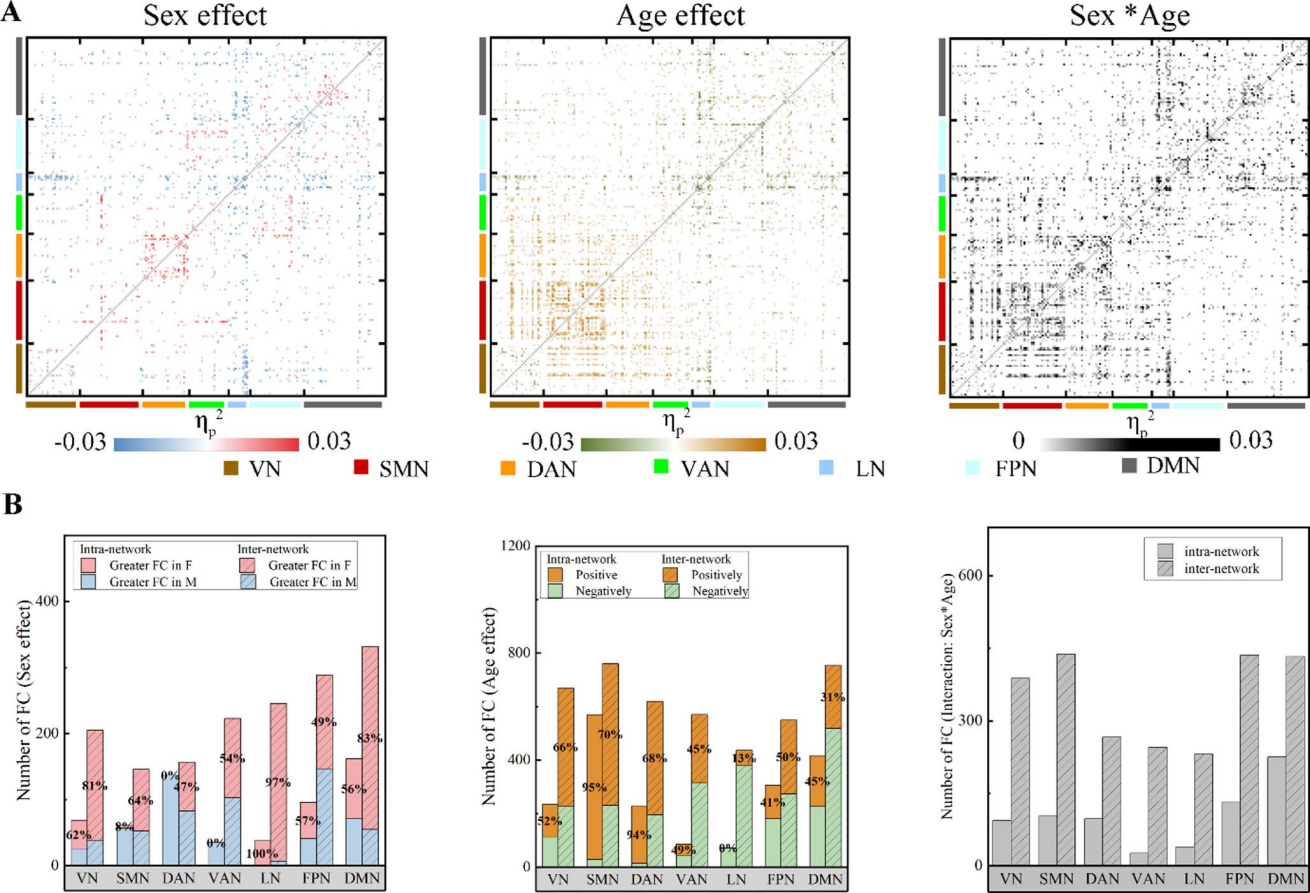
(**A**) The effect of sex, age and their interaction on FC. Sex effect: Negative η_p_^2^ values indicate greater FC in males; positive values indicate greater FC in females. Age effect: Negative η_p_^2^ values indicate a negative correlation between FC and age; positive values indicate a positive correlation. (**B**) Left column: Network-level distribution of main effects of sex on FC. Red denotes greater FC in females (F); blue denotes greater FC in males. Medial column: Network-level distribution of main effects of age on FC. Green indicates a negative correlation between FC and age; brown indicates a positive correlation. Right column: Network-level distribution of the interaction between sex and age. Network abbreviations: VN: visual network. SMN: somatomotor network. DAN: dorsal attention network. VAN: ventral attention network. LN: limbic network. FPN: fronto-parietal network. DMN: default mode network. FDR corrected *p* < 0.05

The mixed-effects model result showed the substantial influence exerted by sex, age, and their interaction on FC (Fig. 2). We observed that during movie watching, females exhibited a larger number of greater FC within the VN, DMN, and limbic network (LN). Males predominated in intra-network connectivity within the ventral attention network (VAN), DAN, and SMN. Furthermore, age-related variations in movie-watching FC patterns were identified. Specifically, FC was positively correlated with age within the VN, SMN, and DAN, while FC was negatively correlated with age within the LN and DMN. Significant sex-age interactions were seen in regions showing a main effect of sex or age. At a sparsity of 0.2 or 0.3, our investigation revealed consistent effects of sex and age on FC akin to those observed at a sparsity level of 0.25 (Fig. S1).

### Movie clip content effects of functional connectivity

**Fig. 3:**
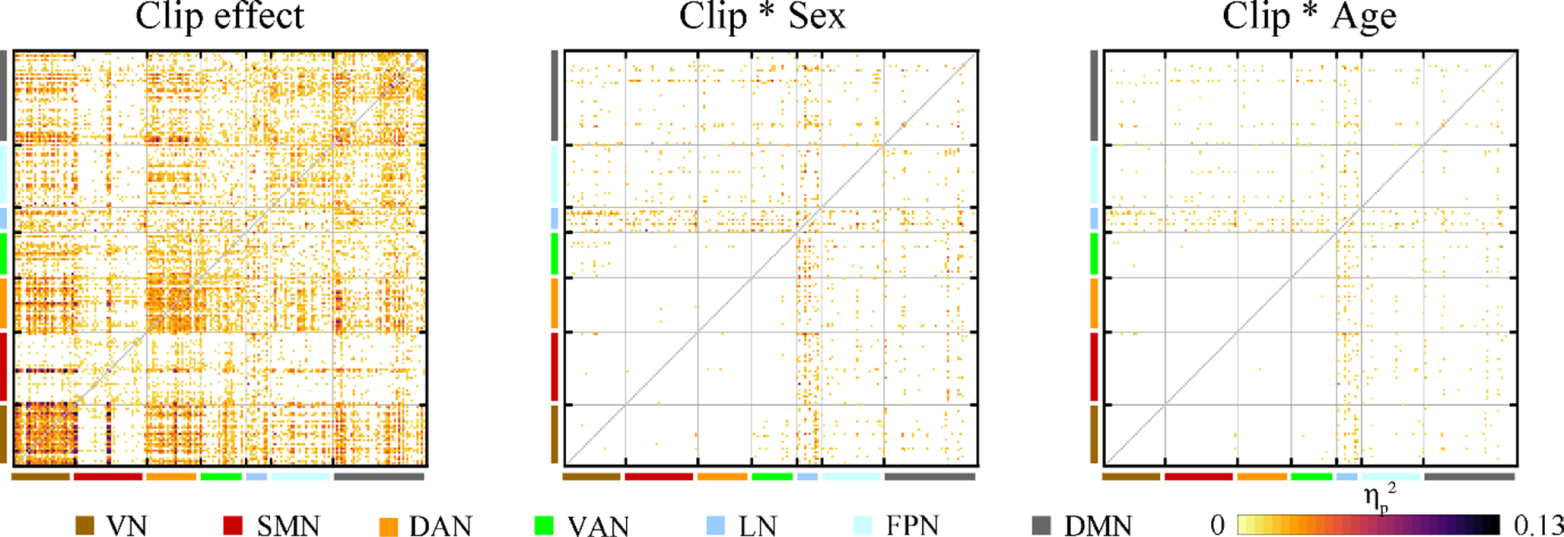
The effect of clip, the interaction of clip with sex, and the interaction of clip with age on FC. VN: visual network. SMN: somatomotor network. DAN: dorsal attention network. VAN: ventral attention network. LN: limbic network. FPN: fronto-parietal network. DMN: default mode network. FDR corrected *p* < 0.05

FC changed in response to variations in the movie clips, with results showing that the effects were most pronounced within the VN. Moreover, the impact of clip content on FC was influenced by the sex and age of the subjects, with interactions primarily observed within the LN and DMN (Fig. 3). Similar results were found for sparsity equal to 0.2 and 0.3. This consistency across varying sparsity choices underscores the stability of our results (Fig. S2).

### Predict movie clips using functional connectivity

**Fig. 4:**
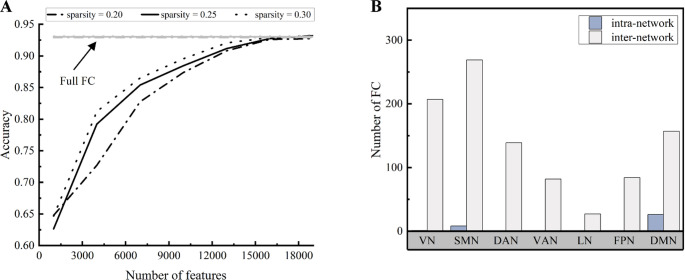
(**A**) Movie clip classification accuracies of SVM based on ACSC-selected features were evaluated at sparsity levels of 0.2, 0.25, and 0.3, respectively. The chance-level accuracy = 6.7%. (**B**) The distribution of the 1000 FC edges with the largest ACSC scores within the seven networks was examined

As the number of selected features progressively increases, a corresponding enhancement in classification accuracy and F1 score is observed. Notably, the ACSC-based feature selection method can achieve the same accuracy/F1 score level as when full FC are used as features, even with only around 15,000 selected features (Fig. 4A; Table 2). Additionally, when categorizing the top 1,000 FC with the highest ACSC scores at the network level, we find that these edges are primarily inter-network connectivity rather than intra-network connectivity, especially in the VN, SMN, DAN, DMN (Fig. 4B). The results obtained from the 10-fold cross-validation were consistent with those from the leave-one-out cross-validation, indicating the stability and reliability of our findings (Fig. S3, Table S1).

**Table 2:** F1 score across different numbers of FC features

Sparsity	Number of features							
	1000	4000	7000	10,000	13,000	16,000	19,000	full
0.20	0.651	0.731	0.830	0.875	0.909	0.927	0.928	0.929
0.25	0.630	0.795	0.856	0.886	0.913	0.928	0.932	0.930
0.30	0.650	0.815	0.868	0.897	0.922	0.930	0.933	0.931

## Discussion

The study investigated the impact of sex, age, and movie content on FC patterns during movie-watching experiences. We observed that sex and age significantly influence FC, with distinct connectivity patterns observed between males and females, as well as across different age groups. Additionally, movie clip content modulates FC, particularly within the visual network, with sex and age differences apparent in FC responses to clip content. This study provides new insights into how demographic factors influence dynamic neural processes during movie-watching, contributing to a deeper understanding of brain-behavior relationships. Moreover, the ability to classify movie clips based on FC patterns highlights the utility of FC as a neural fingerprint, with potential applications in personalized neuroscience and clinical diagnostics.

### Sex and age effects on functional connectivity

Sex and age significantly influence functional activity in the human brain (Zhang et al. 2016). In a comprehensive analysis of a large dataset comprising young adults’ resting state fMRI data, Filippi and coauthors reported that males exhibited greater connectivity in the parietal and occipital lobes, while females showed heightened connectivity in the frontal and temporal lobes (Filippi et al. 2013). Similarly, Allen’s team observed that females displayed stronger intra-network connectivity, while males exhibited stronger inter-network connectivity, particularly within the sensorimotor network (Allen et al. 2011). However, our findings diverge from some aspects of these previous studies, prompting us to hypothesize that these disparities may arise from the distinct neural states between resting and movie-watching conditions. Notably, we observed increased connectivity within the VN, LN, and DMN while watching the movie. This might signify females’ heightened engagement with and processing of the movie’s visual content, as the VN is associated with visual processing (Yang et al. 2015a). Additionally, the increased connectivity within the DMN suggests enhanced extraction of self-referential information during movie content processing (Sheline et al. 2009; Yang et al. 2013), reflecting females’ propensity for more emotional involvement, as indicated by the LN (Rajmohan and Mohandas 2007; Rio et al. 2022). Conversely, males exhibited a predominance of inter-network connections within the SMN and attention network while watching movies. This pattern may stem from males’ selective attention to specific stimuli and sensorimotor integration within the movie content (Stoet 2017), leading to enhanced SMN and attention network connectivity. Connectivity patterns receive hormonal modulation, thus affecting cognitive function. For example, estradiol enhances connectivity between the prefrontal cortex and the hippocampus, supporting females’ verbal memory and emotion regulation (Hara et al. 2015). Conversely, testosterone has been associated with increased connectivity in frontoparietal networks, which are involved in visuospatial processing and executive function (Votinov et al. 2020). The sex differences observed in functional connectivity arise from the interplay of neurobiological, cognitive, and hormonal factors. Investigating these differences is essential for advancing personalized approaches to psychiatric treatment and for refining models of brain function within computational neuroscience.

The influence of age on resting-state FC has been extensively investigated in prior research. However, our study extends this inquiry by exploring the impact of age on FC during movie watching. Although limited by the narrower age range of the dataset (21–35 years), previous lifespan studies have shown that FC within this range exhibits a linear relationship with age (Betzel et al. 2014; Wen et al. 2020). Therefore, our findings suggest that the significant effect of age on FC patterns in response to movie stimuli remains valid. Specifically, elder individuals appear to demonstrate heightened selective attention with the visual and sensorimotor dimensions of the film, thereby manifesting increased FC within networks associated with these functions, such as the VN, SMN, and attention network (Rohr et al. 2018; Vij et al. 2018). In contrast, younger individuals may prioritize alternative cognitive processes, such as memory consolidation and emotion regulation, leading to increased FC within networks like the LN (Carstensen and Mikels 2005; Catani et al. 2013; Kensinger et al. 2007). Lastly, our study revealed a noteworthy interaction between sex and age on FC of the entire brain. This observed phenomenon could be attributed to variations in cognitive reserve and neuroplasticity across different sex and age groups (Lee et al. 2022; Nichols et al. 2021). Sex-specific cognitive strategies and compensatory mechanisms might interact with age-related changes in brain function, leading to distinctive FC patterns.

Numerous brain regions exhibiting the main effects of sex or age also demonstrated significant sex-by-age interaction effects. A previous study using resting-state fMRI revealed sex-specific trajectories of resting-state network development across the adult lifespan. Specifically, males and females showed distinct patterns of connectivity changes in large-scale networks during the transition from early adulthood to late middle age, indicating the presence of sex-by-age interaction effects (Scheinost et al. 2015). Consistent with this, our findings suggest that such interaction effects are also detectable during naturalistic paradigms such as film viewing. Notably, these effects emerged even with relatively brief film clips (64–259 s in duration), underscoring the utility of film-watching fMRI in investigating demographic influences on large-scale brain network dynamics.

### Clip content effects on functional connectivity

FC is subject to modulation by external stimuli, with visual areas playing a crucial role in perceiving and processing such stimuli (Yang et al. 2015a). Li and partners observed an increase in stimulus-driven brain signal energy in visual areas during the presence of stimuli, as evidenced by event-related fMRI experiments (Li et al. 2019). Consistent with these findings, our study confirms that FC exhibits changes based on the content of the movie clip being viewed, particularly within the visual network. Furthermore, we observed sex and age differences in the impact of movie clip content on FC, notably in the LN and DMN. These observations align with prior research indicating that both the LN and DMN are sensitive to sex and age-related developmental changes (Scheinost et al. 2015; Betzel et al. 2014; Wen et al. 2020). Overall, these findings underscore the intricate interplay between sex, age, movie content, and neural connectivity patterns, shedding light on the nuanced mechanisms underlying sex/age/clip differences in brain activity during movie-watching.

### Functional connectivity as a fingerprint of the movie clip

FC emerges as a robust marker for individual discrimination, offering insights into demographic characteristics, mental health status, and cognitive abilities (Dong et al. 2015; Jeong et al. 2015; Rohr et al. 2017; Gong et al. 2019; Shen et al. 2015; Tang et al. 2018). Moreover, FC serves as a nuanced fingerprint of neural responses, delineating the intricate interplay between perceived stimuli and neural performance through advanced neural decoding techniques (Logothetis 2008; Wang et al. 2012). Notably, recent evidence highlights that neural representations are modulated by natural fluctuations in brain activity, especially when processing visual stimuli. Leveraging this understanding, various visual stimuli elicit distinct FC patterns, enabling accurate prediction of image categories with remarkable precision (Liu et al. 2018; Wang et al. 2016). Transitioning this paradigm to the realm of movie-watching fMRI, our investigation unveils the robustness of FC in accurately classifying film clips, underscoring its adaptability as a neural decoding fingerprint across diverse audiovisual stimuli.

The VN, SMN, DAN, and DMN are crucial in classifying movie clips based on their functional connectivity patterns. The VN is central to processing the visual stimuli in movie clips, as it is engaged during visual perception and is primarily activated when viewers focus on visually complex scenes (Yang et al. 2015b). The SMN contributes to the classification process by responding to action-related content, reflecting the network’s involvement in motor processing and sensory input during dynamic scenes (Han et al. 2023; Klingner et al. 2019). The DAN is vital for directing attention, particularly when viewers need to focus on specific aspects of the movie, enhancing the relevance of attention-driven FC patterns in movie classification (Zhao et al. 2022). Finally, the DMN, traditionally associated with self-referential thinking and emotion processing, plays a significant role when movie clips evoke personal reflections or emotional engagement, influencing how viewers connect with the narrative (Sheline et al. 2009; Whitfield-Gabrieli and Ford 2012). The interplay between these networks, as reflected in inter-network connectivity, highlights the complex, integrative nature of cognitive processes involved in movie-watching and underlines their utility as distinctive features for classifying film clips based on functional connectivity.

When utilizing FC as a feature for movie clip classification, we employed advanced feature selection techniques, revealing the efficacy of ACSC feature selection in enhancing classification accuracy with minimal feature redundancy. To achieve precise fingerprinting, it is imperative to capture unique subject-specific information effectively, a feat adeptly accomplished by ACSC through its intricate cross-scan similarity matrix and cost function (Li et al. 2021). Noteworthy, we found that the top 1000 FC features containing the most individual-specific information predominantly comprise inter-network connectivity. This is consistent with the findings of Fan and colleagues, who used FC to predict sex and fluid intelligence and found that inter-network connections contained more weight (Fan et al. 2020).

Our classification framework, combined with the ACSC score for feature selection, highlights the reliability and stimulus-specific nature of FC patterns during naturalistic viewing. Notably, the top contributing connections for classification were predominantly inter-network FCs, particularly involving the VN, SMN, DAN, and DMN. These results underscore the dynamic reconfiguration of large-scale brain networks in response to different cognitive and perceptual demands elicited by movie content. Such findings suggest that the brain’s functional architecture is flexible and context-dependent, supporting the idea that functional connectivity encodes information about external stimuli in a content-specific manner. This reinforces the view of FC as not merely reflecting baseline organization but as an active and meaningful signature of ongoing cognitive processing.

### Limitations and future studies

While we rigorously controlled for sparsity and classifier choosing, some limitations persist. First, we utilized the HCP dataset, known for its high image quality, to investigate the associations between age, sex, and movie-related functional connectivity (FC). However, our findings are exploratory due to the limited age range and sex imbalance. Second, dynamic FC captured more comprehensive brain information than static FC (Jalilianhasanpour et al. 2021; Lurie et al. 2020), potentially providing insights into spatiotemporal dynamics during movie streaming. Future research should explore dynamic FC and improve preprocessing techniques to refine our understanding of brain connectivity in real-world scenarios. Furthermore, evidence suggests that preprocessing steps impact FC (Kassinopoulos and Mitsis 2022) despite efforts to enhance reproducibility using Fix-ICA processed data. These considerations emphasize the ongoing need for refinement and exploration of neuroimaging methodologies. Finally, our study offers preliminary evidence that resting-state FC may serve as a marker for differentiating various film clips. However, the influence of specific movie genre/content, as well as its interaction with age and gender, warrants further investigation.

## Conclusion

This study provided a comprehensive analysis of the effects of sex, age, and movie content on functional connectivity during movie-watching, highlighting the significant role these factors play in shaping neural dynamics. Furthermore, our investigation revealed that FC could effectively serve as a neural fingerprint for classifying movie clips, achieving high classification accuracy using machine learning models. This suggests that FC reflects intrinsic brain activity and dynamically adapts to external stimuli, making it a valuable tool for understanding complex cognitive processes in naturalistic settings. Our findings open new avenues for the application of FC in clinical diagnostics, cognitive neuroscience, and personalized brain-based interventions.

## Electronic supplementary material

Below is the link to the electronic supplementary material.

Supplementary Material 1

## Data Availability

The original fMRI datasets can be publicly accessed at https://db.humanconnectome.org/. Additionally, the code utilized in this study was openly available at https://github.com/yangchengxiao/FC_movie-fMRI_HCP, promoting reproducibility and collaborative engagement in our methodologies.
