# Supplementary material for: The effects of sex and age on movie-watching functional connectivity and movie clip classification
Source: Brain Struct Funct. 2025 Jul 9;230(6):116. doi: 10.1007/s00429-025-02962-0 (PMC12241178; doi:10.1007/s00429-025-02962-0)
Supplement: Supplementary file 1 — Supplementary Material 1 [file 429_2025_2962_MOESM1_ESM.docx]

**Supplementary Material**


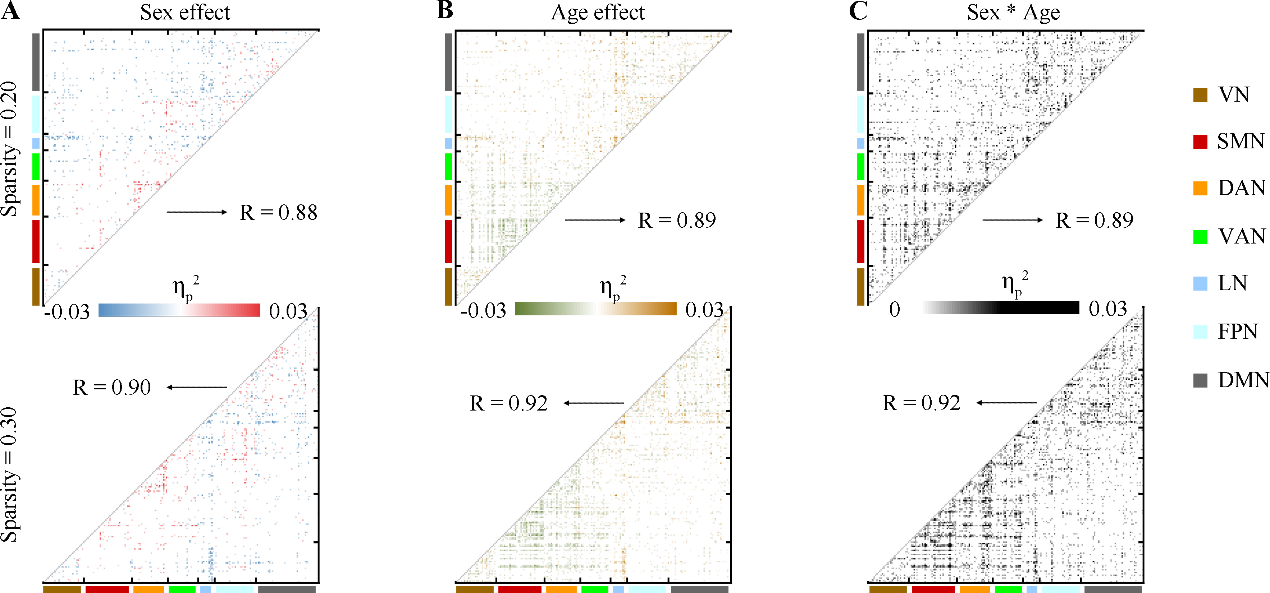


**Fig S1**. The effect of gender, age, and their interaction on FC at sparsity = 0.20 and 0.30. The effects at sparsity = 0.20/0.30 had a high correlation with the results at sparsity = 0.25.


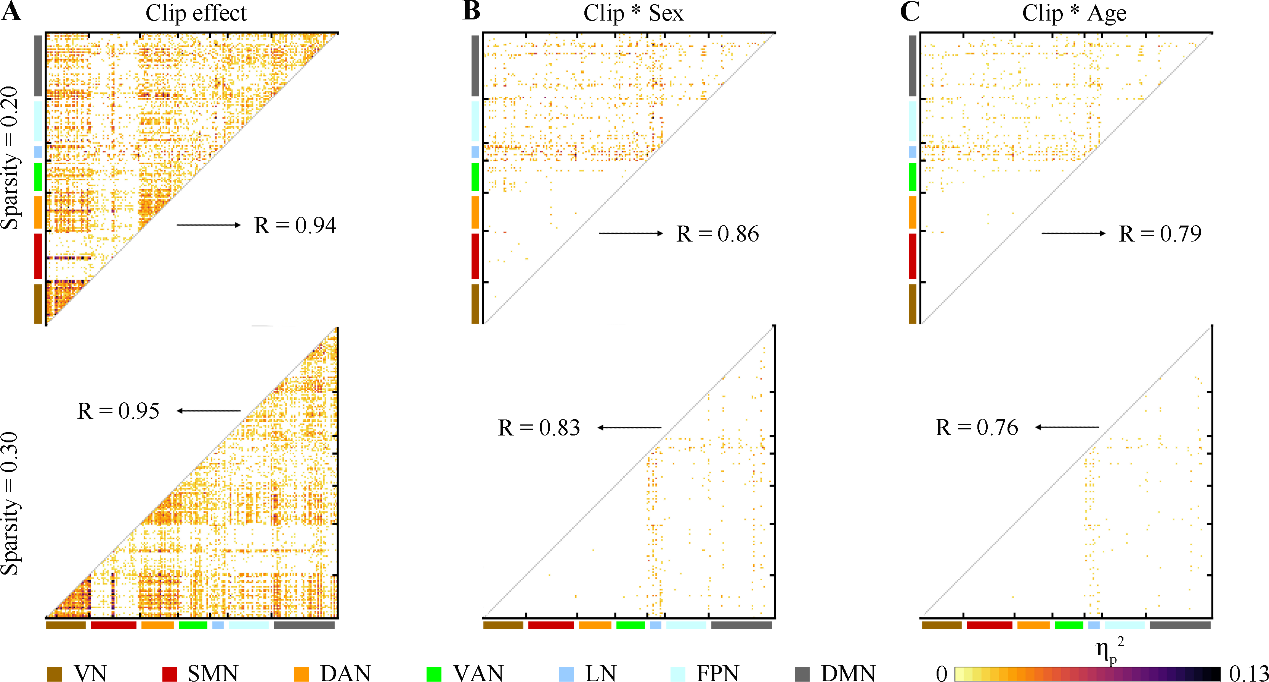


**Fig S2.** The effect of clip, the interaction of clip with gender, and the interaction of clip with age on FC at sparsity = 0.20 and 0.30. The effects at sparsity = 0.20/0.30 had a high correlation with the results at sparsity = 0.25.


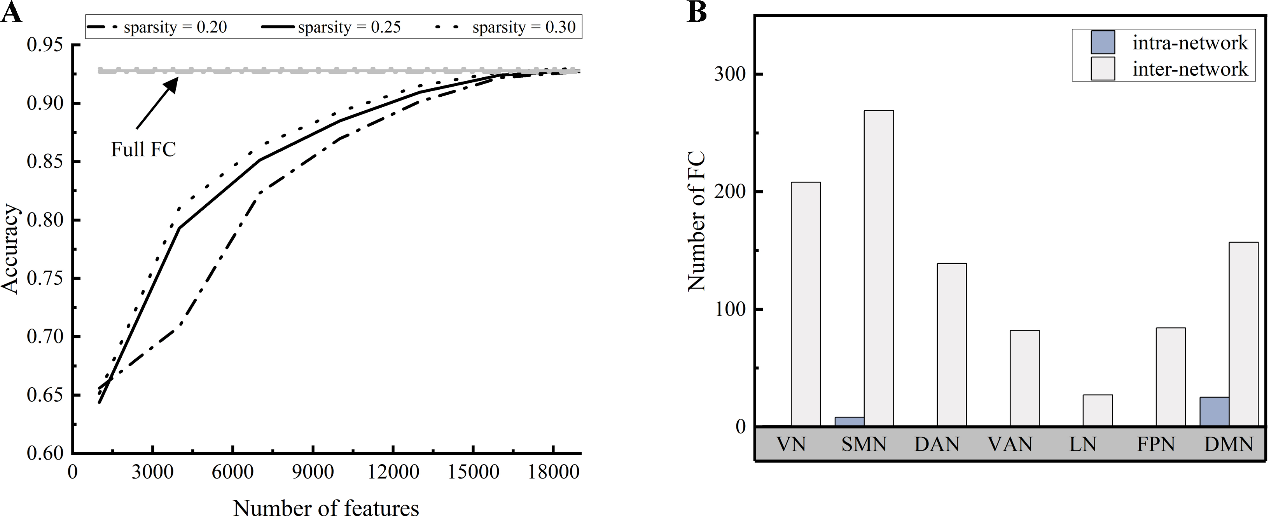


**Fig. S3 A)** Movie clip classification accuracies of 10 folds cross-validation SVM based on ACSC-selected features were evaluated at sparsity levels of 0.2, 0.25, and 0.3, respectively. **B)** The distribution of the 1000 FC edges with the largest ACSC scores within the seven networks was examined.


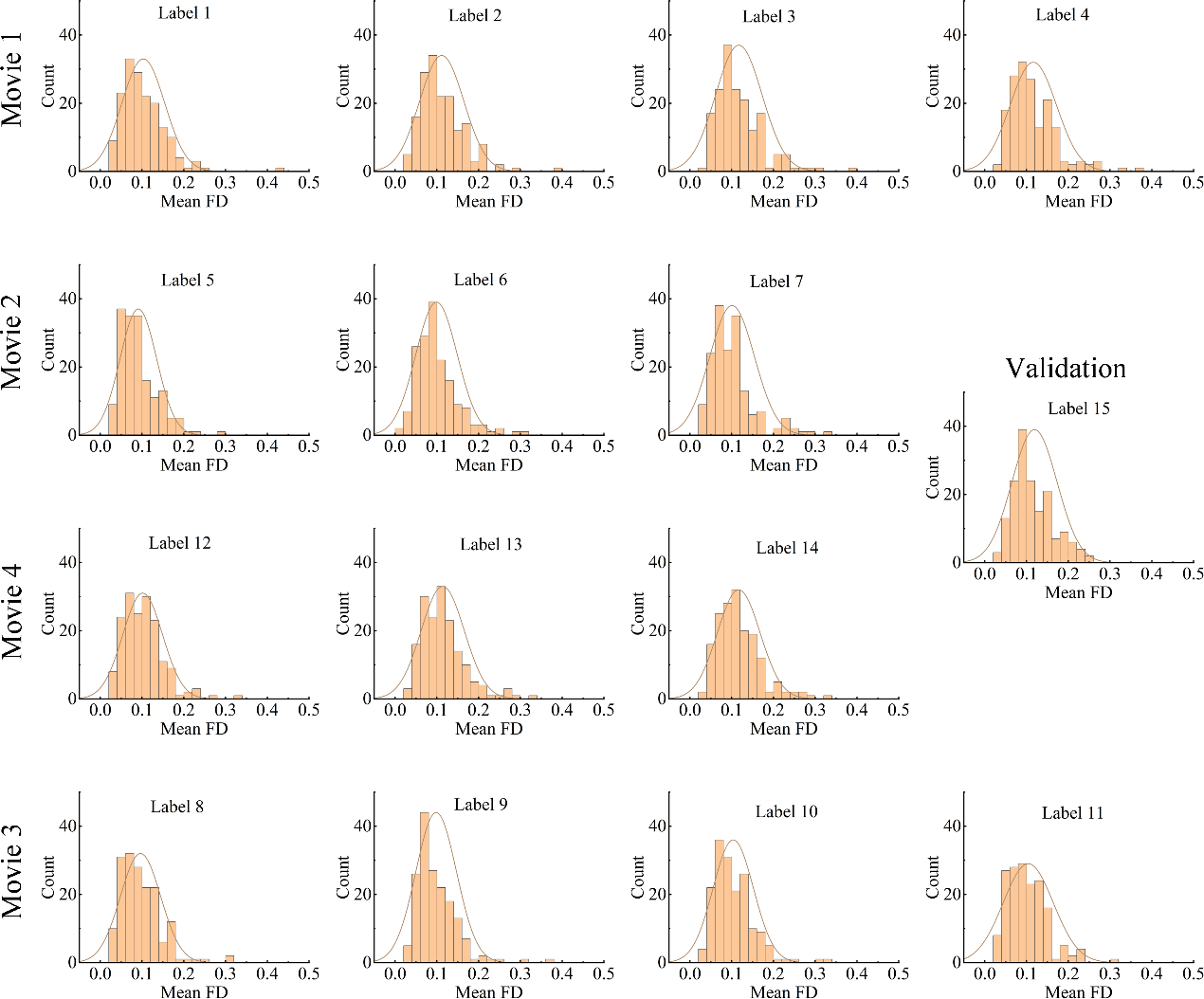


**Fig. S4** Mean framewise displacement (FD) distribution across the 15 clips for 169 subjects. FD were moderate (< 0.5 mm), and the inclusion of head movements as a covariate in the mixed-effects model was effective to control for head movements of the age/sex/clip effect on FC.

**Table S1** F1 score across different numbers of FC features in 10 folds cross-validation SVM.

| **Sparsity** | **Number of features** | | | | | | | |
| --- | --- | --- | --- | --- | --- | --- | --- | --- |
|  | 1000 | 4000 | 7000 | 10000 | 13000 | 16000 | 19000 | full |
| 0.20 | 0.658 | 0.711 | 0.825 | 0.871 | 0.902 | 0.923 | 0.928 | 0.927 |
| 0.25 | 0.646 | 0.795 | 0.853 | 0.886 | 0.911 | 0.925 | 0.929 | 0.928 |
| 0.30 | 0.654 | 0.813 | 0.866 | 0.895 | 0.916 | 0.928 | 0.931 | 0.930 |
